# Supplementary material for: Medical students’ perception of resilience and of an innovative curriculum-based resilience skills building course: A participant-focused qualitative analysis
Source: PLoS One. 2023 Mar 8;18(3):e0280417. doi: 10.1371/journal.pone.0280417 (PMC9994682; doi:10.1371/journal.pone.0280417)
Supplement: S1 File — (DOCX) [file pone.0280417.s001.docx]

# Supporting information

## Glossary terms and abbreviations

3As of Building Resilience: The framework generated by this study, summarizing the perception of students of resilience and of a curriculum-based resilience skills building course. The 3As stand for Awareness, Application, and Appraisal.

MBRU: Mohammed Bin Rashid University of Medicine and Health Sciences

UAE: United Arab Emirates

CoM: College of Medicine

MBBS: Bachelor of Medicine, Bachelor of Surgery, or in Latin: Medicinae Baccalaureus, Baccalaureus Chirurgiae

COVID-19: Corona virus disease of 2019

4M-Model: A framework that suggests the combination of Mindfulness, Movement, Meaning, and Moderator for individual-level interventions aimed at promoting university students' mental health.

MENA: Middle East and North Africa region

OSCE: Objective Structured Clinical Examination

IRB: Institutional Review Board

## Declaration of interest statement

No potential competing interest was reported by the authors.

## Funding

No funding was obtained for this study.

## Ethics approval and participants’ consent

Ethical approval for the study was granted by the MBRU, Institutional Review Board (Reference # MBRU-IRB-2019-021). Written informed consent was obtained from all participants.

## Notes on contributors

BN- contributed to the development of the resilience skills building course; led the delivery of the resilience skills building course; collected the data as part of the reflective essay assignment; contributed to analyzing the data, developing the 3As of Building Resilience framework; contributed to composing the final version of the manuscript; approved the final manuscript.

FO- contributed to the development and delivery of the resilience skills building course; contributed to analyzing the data, developing the 3As of Building Resilience framework; contributed to composing the final version of the manuscript; approved the final manuscript.

AFN- supported in mapping the data as part of the multi-phased data analysis framework; approved the final manuscript.

SH- led the development of the resilience skills building course, assuring its quality; contributed to the delivery of the resilience skills building course; maintained a macro perspective of the research work, contributing to the development of the 3As of Building Resilience framework; approved the final manuscript.

## Acknowledgment

The authors would like to extend gratitude to the following faculty and staff members at the Mohammed Bin Rashid University of Medicine and Health Sciences (MBRU): Dr. Hanan Alsuwaidi, Dr. Reem AlGurg, Dr. Laila Alsuwaidi, Dr. Nusrat Khan, Dr. Adrian Stanley, Ms. Maryam Bukhash, Ms. Sabah Kiran, and Ms. Fatma Kahoor, for their direct or indirect contribution, as per the course study guide, to the development and /or delivery of the innovative resilience skills building course under investigation in the current study. The authors would also like to acknowledge the contribution of the Department of Admissions & Registration (DAR) at MBRU for their support in organizing for the delivery of the course, and keeping record and sharing with the research team the students' registration information.

## Availability of Data

All relevant data are within the manuscript and its Supporting Information files.
