# Supplementary material for: Medical students’ perception of resilience and of an innovative curriculum-based resilience skills building course: A participant-focused qualitative analysis
Source: PLoS One. 2023 Mar 8;18(3):e0280417. doi: 10.1371/journal.pone.0280417 (PMC9994682; doi:10.1371/journal.pone.0280417)
Supplement: S1 Appendix — (PDF) [file pone.0280417.s002.pdf]

جـامـعـة مـحـمـد بـن راشـد
لـلـطـب و الـعـلـوم الـصـحـيـة
MOHAMMED BIN RASHID UNIVERSITY
OF MEDICINE AND HEALTH SCIENCES

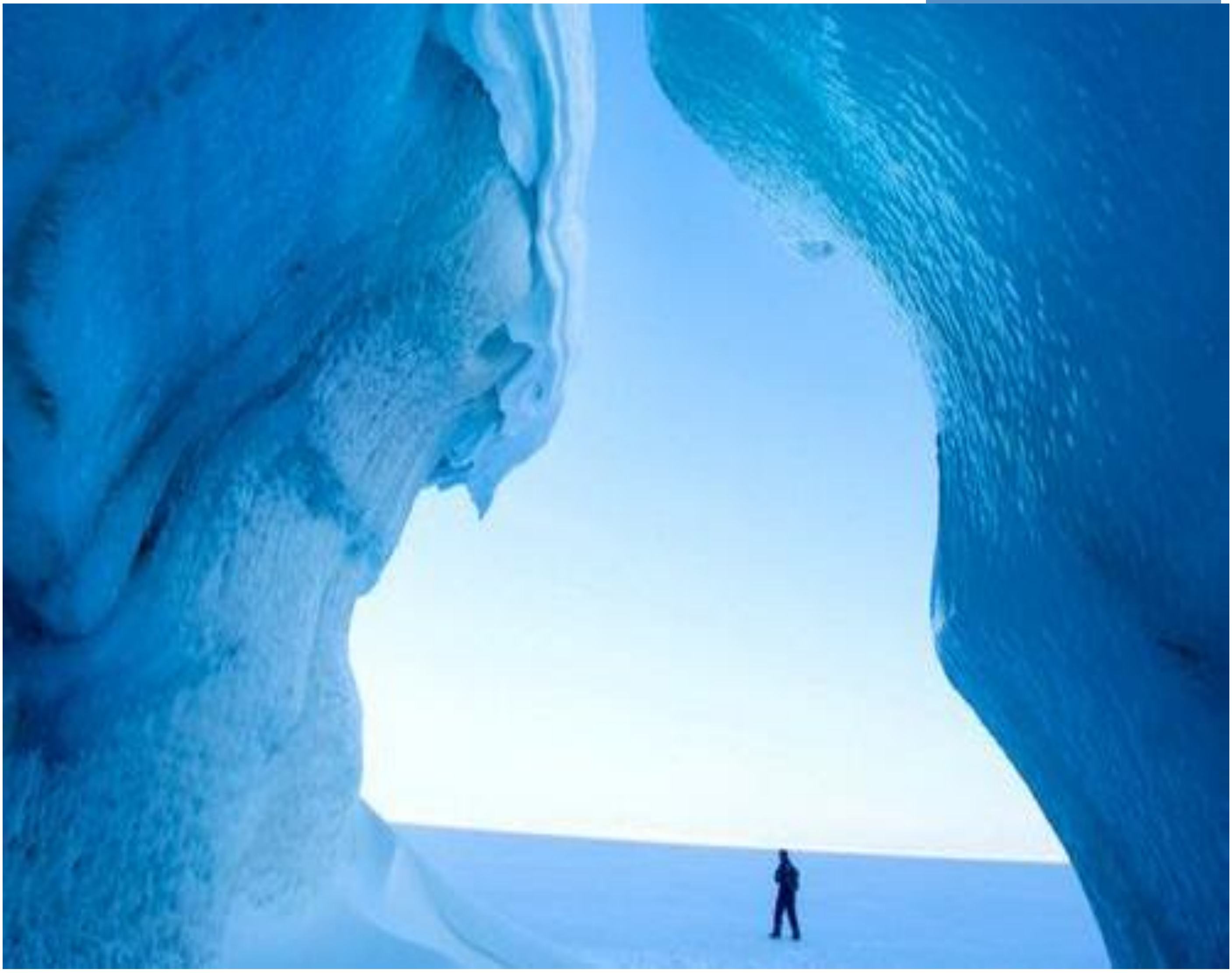

Resilience Skills Building

COURSE GUIDE

YEAR 4

CONTENTS

MBRU FACULTY & ADMINISTRATIVE STAFF 3

COURSE INFORMATION 4

GENERAL INFORMATION 5

LEARNING RESOURCES 5

ASSESSMENT 6

COURSE SCHEDULE (THURSDAY MBRU DAY) 7

Name	Role	Email
Dr. Adrian Stanley	Faculty, Phase III Director<br>Associate Professor of Medicine	Adrian.Stanley@mbru.ac.ae
Professor Samuel Ho	Faculty, Chair-Clinical Sciences<br>Professor of Medicine	Samuel.Ho@mbru.ac.ae
Dr. Laila Alsuwaidi	Faculty, Assistant Dean- Student Happiness & Wellbeing<br>Assistant Professor of Molecular Hematology	Laila.Alsuwaidi@mbru.ac.ae
Dr. Bhavana Nair	Senior Counsellor & Psychotherapist, Student Life	Bhavana.Nair@mbru.ac.ae
Dr. Reem AlGurg	Faculty, Director- Strategy and Institutional Excellence<br>Assistant Professor of Health Policy	Reem.AlGurg@mbru.ac.ae
Dr. Hanan Alsuwaidi	Faculty, Assistant Professor of Family Medicine	Hanan.Alsuwadi@mbru.ac.ae
Ms. Farah Otaki	Senior Specialist- Strategy and Institutional Excellence	Farah.Otaki@mbru.ac.ae
Dr. Nusrat Khan	Faculty, Associate Professor of Psychiatry	Nusrat.Khan@mbru.ac.ae
Ms. Maryam Bukhash	Coordinator	Maryam.Bukhash@mbru.ac.ae
Ms. Sabah Kiran	Coordinator	Sabah.Kiran@mbru.ac.ae
Ms. Fatma Kahoor	Coordinator	Fatma.Kahoor@mbru.ac.ae

Synopsis

This course consists of 6 hours of instruction on building skills for resilience during the first clinical year of the MBBS (i.e., Year 4). The overall objective of this course is to raise awareness about the challenge of stress in the medical students' trajectory and the clinical workplace, and to provide tools for understanding, developing, and deploying resilience skills. By the end of the course, the students are expected to be able to:

- Understand the concept of work-place stress and burnout
- Understand the concept of resilience
- Develop an understanding of the wide variety of tools and techniques that are available to develop and activate resilience
- Comprehend basics of Cognitive Behavioral Therapy
- Demonstrate an understanding of healthy coping strategies
- Understand mindfulness and meditation practices
- Develop knowledge about the skills related to emotional intelligence
- Understand the basic characteristics of mental toughness
- Describe the practice and benefits of gratitude
- Raise self-awareness and perceived self-efficacy
- Enhance one's capacity for emotional self-regulation

In addition, students are meant to develop an attitude of teamwork and self-directed learning through their engagement with the teaching methodology in the course.

Learning Management System (LMS):

The Study Guide will be uploaded in LMS in advance. Details will be added to the course site prior to the first day of each session, and will include presentations, references and resources for that session. Prior to entry into the classroom, students should check the LMS for new information.

Attendance:

All students should attend all classes. Students, who miss more than 20% of the class sessions, will be automatically dropped from the course. Students are required to come on time to each session, as tardiness is not acceptable.

Plagiarism:

The university at MBRU has established strong policies against plagiarism. In submitting your assignments, plagiarism will not be tolerated, and a failing grade will be incurred. Make sure to read the full text given to you in Student Handbook

Note: This Study Guide is subject to minor changes. The student will be informed of any changes in the course content, exam dates, re-scheduling of lectures or tutorials, or any other announcements through the LMS.

Learning Resources

Al Maktoum Medical Library

The Library offers point-of- care resources and medical education databases; quiet areas for individual and group study; and lounges, meeting rooms and an Information Commons. It plays a key role supporting students of MBRU as well as the wider medical community.
The library is accessible at https://www.mbruniversity.ac.ae/en/Library/
Should you have any queries, please feel free to contact the library at library@mbru.ac.ae

Khalaf Ahmed Al Habtoor Medical Simulation Center (KHMSC)

The simulation center is the first comprehensive educational and training facility of its kind in the UAE It is equipped with hospital grade equipment to support health professionals’ education by improving their knowledge and skills in patient care pathway right from Pre-admissions to Discharge. The center showcases realistic hospital environments and clinical skills lab, providing a 'safe' environment for healthcare professionals to learn new techniques, procedures and/or demonstrate appropriate behavior.

This longitudinal course has three components of assessment: attendance requirement, reflection essays, and final OSCE. The OSCEs will be scored and applied to the clerkship rotation courses.

Assessment components	Timescale	Percentage of overall grade	Course outcomes
Attendance	Each session	Must have achieved attendance goal	Pass/Fail
Daily journal of mindfulness practice and reflections	End of session	20%	Formative feedback
Reflection	End of course	30%	Formative feedback
OSCE<br>Must demonstrate knowledge/skills related to resilience	End of course	50%	Pass/Fail

Reflective Essay: Submit a **reflective essay related to your experience with the ideas and skills presented in this course (500 word minimum).** We would like to know what resilience and resilience skills such as meditation and mindfulness means to you. This can include experiences with meditation and/ or activities related to mindfulness in seeing, eating, listening; five senses exercise; PMR, observe your breath exercise; or the body scan. You may also reflect on the skills learnt (CBT, Coping, Personal Boundaries, Emotional Intelligence, Time Management, Mental Toughness). You will receive feedback on your essay
(Minimum 500 words, upload to LMS).

SESSION: Introduction to Resilience and Introduction to CBT
SESSION: Practicing Mindfulness
SESSION: Coping strategies to increase personal resilience
SESSION: Mental Toughness
SESSION: Emotional Intelligence
SESSION: Time Management

Venue: *12.00 – 13.00Hrs;* ***Simulation Center LH 6 (GROUP A) & LH 7/8 (GROUP B)*****;** ***Right Wing, 3rd Floor***
NOTE: students are split into two small groups, A and B with the schedules for each of these groups indicted below.

Session Number	Session	Faculty
**1**	**Introduction to Resilience**	
	Group A: **Introduction to CBT**<br>Group B: **Mental Toughness**	A: Dr. Bhavana Nair<br>B: Professor Samuel Ho
**2**	Group A: **Practicing Mindfulness**<br>Group B: **Emotional Intelligence**	A: Dr. Bhavana Nair<br>B: Dr. Laila Al Suwaidi, Farah Otaki
**3**	Group A: **Coping strategies to increase personal resilience**<br>Group B: **Time management strategies**	A: Dr. Bhavana Nair and Farah Otaki<br>B: Dr. Reem Al Gurg, Professor Samuel Ho
**4**	Group A: **Mental Toughness**<br>Group B: **Introduction to CBT**	A: Professor Samuel Ho<br>B: Dr. Bhavana Nair
**5**	Group A: **Emotional Intelligence**<br>Group B: **Practicing Mindfulness**	A: Dr. Laila Al Suwaidi, Farah Otaki<br>B: Dr. Bhavana Nair
**6**	Group A: **Time management strategies**<br>Group B: **Coping strategies to increase personal resilience**	A: Dr. Reem Al Gurg, Professor Samuel Ho<br>B: Dr. Bhavana Nair and Farah Otaki

SESSION: INTRODUCTION TO RESILIENCE

OBJECTIVES: After participation in the session, learners should be better able to:
- Define foundational concepts relevant to wellness and mindfulness
- Keep a daily practice journal

INSTRUCTIONAL MATERIALS
- Online PowerPoint Presentation
- Flipcharts

CLASS PREREQUISITE
- Complete online Introductory session

Time Segment Description

0-15 Session Introduction: Introduce the session instructor and the participants; present the curriculum and session objectives, expectations, and evaluation methods.
Present foundational concepts, with attention to: stress and anxiety, burnout, and resilience.
Session Closer: Introduce the concept of keeping a daily practice journal

SESSION: INTRODUCTION TO CBT

OBJECTIVES:
After participation in the session, learners are expected to be able to:
- Become more aware of the connection between thoughts, emotions and behavior and how they influence each other
- Identify automatic thoughts and how they can influence their behavior
- Comprehend & define terms like CBT, cognitive distortions, and cognitive reframing
- Understand how to alter emotions by changing dysfunctional thoughts and behaviors
- Learn to ground themselves in the present moment using breath as a medium
- Keep a daily practice journal

INSTRUCTIONAL MATERIALS
- PowerPoint Presentation
- Worksheets

CLASS PREREQUISITE
- Present research component with verbal consent

Time Segment Description

0-10 Session Introduction: Present session objectives and introduce the concept of CBT and how it relates to medical students. Introduce the concept of cognitive model and explain thought stopping and reframing linked to the model

10-35 Explain cognitive distortions and do an exercise on identifying personal distortions

35-45 Exercise: Teach students to identify automatic thoughts and train them to reframe a negative thought

45-55 Introduce the concept of keeping a daily practice journal and teach them a quick breath meditation which they can use just before they go to sleep.

55-60 Session Closer: Review session objectives and discuss prerequisite assignments for next session.

Suggested reading/watching for next session
- Jon Kabat-Zinn’s 9 attitudes – mindfulness
  https://www.youtube.com/watch?v=2n7FOBFMvXg
- Ted Talk on ‘The power of Mindfulness’ – what you practice grows
  https://www.youtube.com/watch?v=IeblJdB2-Vo

- What are the benefits of Mindfulness? Daphne M. Davis and Jeffrey A. Hayes (2012). https://www.apa.org/education-career/ce/mindfulness-benefits.pdf

SESSION: PRACTICING MINDFULNESS

OBJECTIVES:

After participation in the session, learners are expected to be able to:

- Be able to comprehend the main concept of mindfulness
- Understand the importance of developing positive attitudes for sustained self-care to handle the pressure of being a medical student
- Incorporate evidence-based mindfulness techniques to trouble shoot challenges faced in clinical settings
- Learn to practice meditation using Body Scan/Progressive Muscle Relaxation/ Breath

INSTRUCTIONAL MATERIALS

- PowerPoint Presentation
- Bean Bags
- Dates

Assignment:

- Maintain a journal on meditation practices and mindfulness

Time - Segment - Description

0- 5 Session Introduction: Present the session objectives; explain how this session fits into the larger curriculum, and introduce the concept of mindfulness

5-15 Present evidence supporting the effectiveness of meditation and mindfulness, including physiologic and mental health changes. Discuss the 9 attitudes of Jon Kabat Zinn

15-25 Eating dates mindfully- activity & discussion (in pairs)

25-40 Group Discussion: present experiences from the clinical setting. Discuss by linking it to how mindfulness can help defuse challenging situations

40- 55 Mindfulness practice session: Progressive Muscle Relaxation with body scan and breath meditation

50-55 Reflection group discussion: Reflect on the experience

55-60 Session Closer: Review session objectives; discuss prerequisite assignments for next session

Additional Readings:

- Read Staying Sane- addressing the growing concerns of mental health in medical students https://www.amsa.org/2015/09/08/staying-sane-addressing-the-growing-concern-of-mental-health-in-medical-students/

- Jessica Slonim, Mandy Kienhuis, Mirella Di Benedetto & John Reece (2015) The relationships among self-care, dispositional mindfulness, and psychological distress in medical students, Medical Education Online, 20:1, 27924, DOI: 10.3402/meo.v20.27924

Assignment:

- Keep journal on meditation practices and mindfulness

SESSION: COPING STRATEGIES TO INCREASE PERSONAL RESILIENCE

OBJECTIVES:

After participation in the session, learners are expected to be able to:

- Comprehend the two major coping styles (Emotion-focused, problem focused)
- Become more insightful of themselves as future doctors
- Understand how to set personal boundaries for self-care
- Become aware of how self-care can improve personal life and patient care
- Learn to use gratitude as a coping skill to strengthen resilience

INSTRUCTIONAL MATERIALS

- Short Power Point presentation
- Worksheets
- Glass jars/stationery

Time Segment Description

0-5 Session Introduction: Present the session objectives; explain how this session fits into the larger curriculum

5-15 Introduce the two different styles of coping. Students engage in identifying personal coping into the two styles.

15-25 Group discussion: Students reflect on coping styles within the larger group.

25-40 Introduce the concept of the self & setting personal boundaries

40-50 Explain the concept of Gratitude and mental subtraction. Do the 'gratitude jar' exercise.

55-60 Session Closer: Review all the previous 3 session objectives; answer questions regarding the sessions

Additional readings:

• The Fallacy of Chasing After Work-Life Balance"

Assignments:

• Maintain a journal on skills learnt

SESSION: MENTAL TOUGHNESS

OBJECTIVES:

After participation in the session, learners are expected to be able to:

• Define mental toughness and be able to describe the need for it

• Outline personal characteristics of mental toughness and how to measure this

• Determine mental toughness score and create a mental toughness training plan

INSTRUCTIONAL MATERIALS

• PowerPoint presentation

• Flip charts and posters

Time Segment Description

0 -5 Session Introduction: Present the session objectives; explain how this session fits into the larger Curriculum

5-15 Defining definitions of mental toughness and the challenges

10-35 Introduce methods for testing mental toughness

35-50 Determine personal mental toughness score and design a training plan

50-60 Session Closer: Review session objectives; discuss assignment of final reflective essay for next session; answer questions regarding the daily practice journal and following up on the creation of the individualized wellness plan.

Additional readings:

• "Mindset-Changing the way you think to fulfill your potential" by Dr. Carol S. Dwek

• "Grit-Why passion and resilience are the secrets to success" by Dr. Angela Duckworth

Assignment:

• Maintain a journal on skills learnt

SESSION: EMOTIONAL INTELLIGENCE

OBJECTIVES:

After participation in the session, learners are expected to be able to:

• Understand the meaning and components of emotional intelligence

• Illustrate methods for emotional regulation

• Discuss essential aspects of communication skills

INSTRUCTIONAL MATERIALS

• PowerPoint Presentation

Time Segment Description

0-5 Session Introduction: Present the session objectives; explain how this session fits into the larger Curriculum

5-10 Journal entries and discussion

10-15 Group Discussion Students reflect on any changes that have occurred following a regular meditation practice

15-55 Present different aspects of Emotional Intelligence

55-60 Session Closer: Review session objectives; discuss prerequisite assignments for next session; answer questions regarding the daily practice journal and following up on the creation of the individualized wellness plan

Assignment:

Maintain a journal on skills learnt

SESSION: BUILDING RESILIENCE WITH TIME MANAGEMENT

OBJECTIVES:

After participation in the session, learners are expected to be able to:

- View building resilience from the perspective of time
- Recognize how people embody resilience
- Learn techniques to foster resilience, and better manage one’s time and priorities

INSTRUCTIONAL MATERIALS

• PowerPoint presentation

Time Segment Description

0 -5 Session Introduction: Present the session objectives; explain how this session fits into the larger curriculum

5-10 Journal entries and discussion. Reflect on how one can manage time and be in control of it

10-35 Offer a new perspective on resilience by connecting time management skills with resilience

35-50 Introduce time management skills and approaches

50-60 Session Closer: Review session objectives; discuss prerequisite assignments for next session; answer questions regarding the daily practice journal and following up on the creation of the individualized wellness plan

Assignment:

• Work on the final reflective essay
