# Supplementary material for: Medical students’ perception of resilience and of an innovative curriculum-based resilience skills building course: A participant-focused qualitative analysis
Source: PLoS One. 2023 Mar 8;18(3):e0280417. doi: 10.1371/journal.pone.0280417 (PMC9994682; doi:10.1371/journal.pone.0280417)
Supplement: S1 Data — (DOCX) [file pone.0280417.s003.docx]

**Reflections on Mindfulness (A^3)**

| **Theme** | **Categories** | **Exemplars** |
| --- | --- | --- |
| Awareness/ Understanding/ Meaning | Personal interpretations of resilience  **24** | 1M: “…resilience to me refers to willpower. The ability to persevere when times get tough…resilience is what gives people the psychological strength to cope with stress and hardship. It is the mental reservoir of strength that people are able to call on, in times of need, to carry them through without falling apart…”  2F: “…to me resilience is more than a practice performed consciously in life, it is more an unconscious attribute that leads into a calmer, more peaceful state. I find resilience to be the ability of a person to adapt to a new environment… I find that resilience plays a huge role in my day-to-day life, I mostly do it unconsciously without thinking of the act, but it still manages to lift-up my spirit and get me back to a state of peace and tranquility…”  4F: “…I realized that it is not about ‘not experiencing hardships’… Life is hard. Yet, resilience enables you to bounce back from the problem and not remaining ‘stuck’…”  5F: “…to be resilient is to withstand life’s challenges and grow stronger from them…”  6F: “…resilience is the ability to bounce back from difficult life experiences… ‘toughness’ is not a single attribute of an individual, but rather a result of numerous internal qualities and external resources… key resilience skills are self-awareness, attention, letting go physically and emotionally, and accessing and sustaining positive emotions...”  8M: “…resilience, in my life, was embodied in my ability to adapt to a constantly changing environment…”  10M: “…is the capacity of a person to recover quickly from difficulties encountered in life, or to be able to accommodate and adjust to obstacles… resilience enables people to establish the psychological strength that they need to cope with stress and events encountered in life…”  12F: “...resilience to me is when you ‘fall down seven times, stand up eight’. In other words, it is the persistent, conscious decision to keep going, despite all the challenges that you might face. It means that you should stand back up no matter how hard you get pushed down and you should keep going no matter what life throws at you… enabling people to marshal the strength to prosper through the difficult situations they may face in life… Resilient people have a compelling reason to get out of bed in the morning every day as they are committed to their life and goals…”  13M: ”...resilience to me is ‘adapting’, or changing one’s characteristics to become adjusted to a new condition, or even modifying a habit to make it more suitable for the current situation in order to respond to a scenario and such a change may be merely a passive response to an event. For instance, ‘adapting’ to failure may overcome the present obstacle, though it does not mean the person is now more capable to adapt to such a scenario in the future; as what has happened is an adjustment to resolve the current problem, and not an active effort to take this event as a learning experience in order to overcome any such similar event in the future…. as humans, we are constantly subjected to stress in different forms and degrees, and being resilient means being able to recover to our original state after being impacted by a stressor… resilience can be seen as an active, continuous process, rather than a simple adaptation or adjustment following a stress. It is important first of all to develop a force which will resist pressure from the outside world, and even when such pressures are capable of deforming us, resilience will allow us to immediately recoil back to our original self.”  14F: “…resilience enables us to keep moving forward with intention and meaning rather than only for the sake of moving forward by helping us to integrate our experiences and thus re-evaluate our approaches every living moment…”  15F: “…I would personally define psychological resilience as the innate skill to mentally/emotionally overcome a crisis…..it is a mental process that protects a person from the negative effects of external pressure… it allows people to remain level-headed during a crisis and to be able to move on from the incident minimizing long-term negative implications…”  16F: “…as humans we are built to be resilient, to bounce back and heal from whatever life throws at us, whether its physical pain like the flu or a broken bone, or whether its emotional pain like losing a loved one or failing at something…”  17F: “…resilience, to me, is the ability to keep striving and never rest your head on the pillow of surrender. It is knowing that a failing grade is not a failure, rather, giving up on changing that failing grade is a failure. It is knowing that failing to make the right decision at some point in your life is not failing, rather, it is the inability to quit the feelings of guilt, defeat, and surrender… resilience is the ability to accept that one wrong decision weighs almost nothing compared to your strengths and your abilities to believe in yourself and think of changing that minute decision into something great. It is ‘fall down seven times, stand-up eight’. It is knowing when to stop, acknowledging one’s limits, and being aware of when to take a break; it is all about knowing how much you can and cannot do…”  18F: “…resilience is a personal characteristic, a tool of self-reliance and self-preservation, that can be actively cultivated to equip individuals with the right coping strategies to weather any adversity in an enriching manner. In essence, it enables individuals to respond to unfavorable situations healthily…”  19F: “…resilience is not a skill that we are born with. It is a characteristic that one learns to develop and build slowly over time, a set of skills that we improve throughout our life…resilience would be ‘fall down seven times, stand-up eight’. In other words, it, to me, is the persistent, conscious decision to keep going, despite all the challenges that you might face. It means that you should stand back up no matter how hard you get pushed down and you should keep going no matter what life throws at you… practicing resilience helps us in looking at our setbacks and mistakes as lessons in which we can learn from and as opportunities in which we can grow and prosper from. It also helps us in maintaining a positive outlook of the future and envisioning brighter days to come ahead…”  20F: “…psychological strength is just as crucial as physical strength when it comes to coping with hardships. Resilience is the mental reservoir of strength that grants us that psychological strength to push through the endless stressors that life hands us whenever need be… It is inevitable that everyone will deal with changes and losses, minor and major, thus, how we deal with these problems can play a significant role in not only the outcome but also the long-term psychological consequences. The latter is where resilience plays a role. It is the ability to withstand stress as well as to adapt and overcome changes. When put through hardships that is where our resilience is put to test, especially if we get into a situation where we are not in control or if the situation was not expected, and when a person is resilient, they will be able to accept the stressor, bounce back from every low, and keep going albeit it is widely different from resisting a hardship and not responding to stressors…”  22F: “…resilience to me means being adaptable to whatever curveballs life throws at you, it is the ability to continue with your day and still be productive even when inconvenience occurs…”  26F: “…resilience to me is the ability to recover and bounce back from a difficult situation. This is a skill that requires a stable state of mind and is thus improved with the help of various techniques such as meditation and journaling…”  29F: “…resilience is the act of getting back up every single time you have been pushed down. It is the act of carrying your weight back up along with the mistakes that you have made that led you to fall. The art, however, lies in one’s ability to defer the negativity of their own mind and that of the people who surround them. Instead of standing up with anger and fear, one would stand up with hope and determination to be better and to learn from their mistakes…”  31F: “…it is the ability to withstand stress as well as to adapt and overcome changes. When you are put through hardships that is where our resilience is put to test, especially if we get into a situation where we are not in control or if the situation was not expected, and when a person is resilient, they will be able to accept the stressor, bounce back from every low, and keep going albeit it is widely different from resisting a hardship and not responding to stressors… the key factors to resilience include having a positive outlook, spirituality, active coping, learning, finding the meaning in a difficult situation, and accepting one’s own personal limits. Having the perspective of seeing the ‘glass half full’ is crucial to resilience because understanding that setbacks are a part of life can change a person’s mindset, and help them remain open to new situations and become adaptable to change since problems are not always avoidable…”  32F: “…resilience is the person’s ability to cope with life stressors and prevent anxiety; through developing emotional and behavioral mechanisms to deal with the undesirable outcomes of these stressors… resilience for me means the ability to adapt to challenges in life, it is the plasticity and flexibility that allows me to overcome hard feelings and tough moments. Resilience is understanding the importance of self-care including physical and mental health. It is the small steps that are done every day and that lead to bigger outcomes…”  33F: “…I see resilience as how each and every person faces their own battles and how they come out of those battles, sometimes the methods that we use are effective in reducing the stress of the battle, other times it is not as effective and that is okay…”  34F: “…to me, resilience means coping. Coping badly with the busy schedules ahead, the huge mental strain, and sometimes the lack of social life can leave you overwhelmed and slowly lead to depression…”  37M: “…you do not have to think of the past or the future when you meditate…” |
|  | Perceived associations  **11** | 1M: “…social support is another critical variable that contributes to resilience. Mentally strong people tend to have the support of family and friends who help bolster them up in times of trouble… mindfulness means living in the present moment… with acceptance and without judgment…mindfulness and meditation are different... However, they are connected. While mindfulness strengthens and enhances meditation, meditation nourishes and expands mindfulness…”  6F: “…I think mindfulness is a key tool for training attention, and a particular kind of attention: present-moment awareness that is receptive, accepting, kind, and appreciative… reflection is also important for mindfulness, it permits us to venture outside the circumstance, considering ourselves to be the onlooker as opposed to the person in question, and frees us up to different perspectives. By widening our point-of-view, we are better ready to manage those reactions that are ruminous…”  10M: “…supplementing resilience to mindfulness leads to better outcomes and management of ones’ hardships…”  13M: “…mindfulness and resilience may not be synonyms, but are definitely not antonyms either, rather they are complementary as achieving the skill of mindfulness will allow an individual to obtain the character trait of resilience… in order for a person to be resilient, they must be able to practice mindfulness, as this is the most important skill that has to be achieved in order to develop true and functional resilience…”  15F: “...I recognize the effect of resilience in me from previous memory, more as a process than an actual trait found in individuals. This process is an individual continuous coping strategy procured by protective environments and stable social relationships which makes resilience more likely to occur. Another variant that contributes to resilience is emotional intelligence which seems a very important concept when it comes to building resilience and practicing gratitude...”  17F: “…mindfulness and meditation – the quintessence of building one’s resilience – have been prevalent since time immemorial, albeit in their varied art forms, that have evolved over time to suit the hustling, bustling lifestyle of the present generation… mindfulness is the practice of being truly and wholly present in one’s reality. It is appreciating reality in stop-motion – pausing to bring awareness to and process one’s thoughts, emotions, and sensations in each frame i. e. every present moment…”  20F: “…the definition of mindfulness that I like to go by is that it is defined as non-judgmental awareness of our thoughts, feelings, sensations, and surroundings. Although, the latter isn’t the easiest task that a person might assign themselves to but in order to practice mindfulness we can train ourselves to stop rehashing the past in our thoughts or imagining the future and actually be in tune with what we sense presently…”  22F: “...the journey of resilience and mindfulness is not a sprint, rather it is a marathon. I would like to think that resilience starts with mindfulness, to be resilient in difficult situations requires a moment of reflection and awareness to your response to the obstacle…”  23M: “…mindfulness to me is all about gaining control over your thoughts by organizing this “chaotic storm” into a more manageable and most importantly peacefully flowing “river”, creating a space for your unconscious to focus on the tasks at hand and ultimately making the most out of very little brain power…”  29F: “…mindfulness is one of the resilience skills that allows someone to be fully present and aware of their surroundings and actions in a calm and focused manner. In other words, it also means it is one’s ability to remain calm, calm in the sense that they do not act impulsively or rage with anger after a distressing event…”  33F: “…mindfulness can help build resilience and manage stress…” |
|  | Perceived relevance  **21** | 1M: “…this is particularly pertinent when studying medicine as the study load can at times become over burdensome…”  2F: “…as a 4^th^ year medical student, the meaning of resilience to me changes with each stage of my medical career…”  3F: “…prior to entering the field of medicine, I was sheltered from what a stressful and demanding lifestyle could manifest into. Upon embarking this journey, I found myself seeking the oblivion of sleep, or occasionally Netflix, whenever I encountered a stressful situation. Unfortunately, I have concluded that medical school can sometimes garner itself as burn out and anxiety galore. There reached a point, where my circadian rhythm could no longer give into my form of stress relief that I decided to invest my time researching the world wide web for coping mechanisms. To be frank, upon encountering mindfulness I was nothing short of skeptical and somewhat doubtful…”  4F: “…It helps in slowing down our fast-paced lives and minds, and it draws our attention to the present moment…”  5F: “…as future physicians, we need to become resilient. We will inevitably come across many challenges on a regular basis. It may be easier to shy away from these challenges, but with resilience, we are able to face the challenges head-on, and ‘exit’ the corresponding situation stronger and wiser than before…”  6F: “…as future physicians, it is important to promote mindfulness, while using it in our daily lives…”  9F: “…as medical students, we go through a lot of stress and anxiety; mindfulness is one of the most efficient ways to reduce stress and go about our days in a relatively good mood… I think mindfulness is a vital technique that helps with focusing …welcoming and appreciating your surroundings…”  10M: “…as a medical student, it is no surprise that resilience is quite important in order to be able to cope with the stress and anxiety of academia which can be overwhelming… practicing mindfulness and building resilience are imperative to avoid burning-out and to maintain one’s wellbeing…”  12F: “…resilience is a valuable skill to develop because it gives us the strength that we need to overcome challenges we face in our daily life and enables us to maintain balance in our life… practicing resilience helps us look at our setbacks and mistakes as lessons and as opportunities to learn from. It helps us to grow, prosper and maintain a positive outlook of the future... as a medical student, I believe that practicing mindfulness and enhancing resilience are beneficial tools that help mitigate stressors and burnout, and improve the wellness of medical students. I find this to be especially true for clinical experiences in medical school as we encounter new stressors during this time, becoming at risk of burnout. Transiting from the classroom to the clinical environment holds a lot of new challenges and can trigger many mental health conditions, and practicing resilience could play a protective role in clinical training and can help improve our professional quality-of-life during our clinical rotations…. the medical profession also involves inherent unpredictability that demands future doctors to be adaptable, therefore, resilience is one of the most valuable traits a physician can have to enhance psychological strength to cope with stressful situations in our life. Building resilience in doctors and healthcare workers has a long-term effect on the quality of health care, and strongly impacts the way in which patients are cared for…”  13M: “…as medical professionals who believe ‘prevention is better than cure’, this may translate into building resilience. as well… as students, we must focus on becoming resilient by developing the emotional and mental skills necessary to prevent outside pressures from hindering our potential, even before we are faced with stressors…”  14F: “…resilience, regardless of the technique you chose to practice, is an essential set of skills in the modern world, and it will probably dominate our emotional intelligence reservoir for a significant time in the future. It is therefore of prime importance to invest a shred of your daily routine to develop these mighty skills...”  15F: “…circumstances that medical students will need to face in the near future require a strong resilient nature to be able to progress and flourish in our respective lives…”  16F: “…I learned to allow myself to feel discouraged sometimes but not to remain discouraged. Resilience is not about masking pain and pretending everything is peachy, we are humans after all, not a machine. It is not how you feel in the moment that defines you, it is how you choose to overcome it and stand back up…”  17F: “…resilience is the reason we hold onto our strengths and improve our weaknesses… resilience is the reason I am on my feet every day, why I continue to strive and work harder…”  18F: “…life as a professional in the healthcare sector can be remarkably arduous and demanding, physically and psychologically. Enduring long working hours, day-to-day exposure to patient trauma and patient deaths, the looming overhead fear of contracting infectious diseases are just few of the numerous stressors that tie into how truly unpredictable a regular work day can be for a physician. These stressors are more than sufficient to induce detrimental psychological reactions, and result in burnout… resilience has never been more relevant among healthcare professionals, given the current, unprecedented pandemic…’  19F: “…resilience is a valuable set of skills to develop because it gives us the strength that we need to overcome challenges we face in our daily life enabling us to maintain balance. People who possess resilience do not see life through rose-colored lenses, or have a life free of stressors and difficulties. They understand that they may face some challenges and that setbacks happen and that sometimes life does not go the way that they want it to go. They still go through painful experiences and difficulties but their outlook towards life allows them to work through these challenges and adapt to them. Resilience enables people to marshal the strength to prosper through the difficult situations they may face in life… resilient people have a compelling reason to get out of bed in the morning everyday as they are committed to their life and goals… as a medical student, I believe that practicing mindfulness and enhancing resilience are beneficial tools that help mitigate stressors and burnout which improve the wellness of medical students. I find this to be especially true for clinical experiences in medical school as we encounter new stressors during this time and become at greater risk for burnout. Transiting from the classroom to the clinical environment holds a lot of new challenges and can trigger many mental health conditions, and practicing resilience could play a protective role in clinical training and can help improve our professional quality of life during our clinical rotations… the medical profession also involves inherent unpredictability that demands future doctors to be adaptable, therefore, resilience is one of the most valuable traits a physician can have. Building resilience in doctors and healthcare workers has a direct effect on the quality of health care and medical care, and strongly impacts the way in which patients are cared for...”  20F: “…the key factors to resilience include having a positive outlook, spirituality, active coping, learning from and making meaning of a situation, and accepting your own personal limits. Having the perspective of seeing the ‘glass half full’ is crucial to resilience because understanding that setbacks are a part of life can change a person’s mindset, and help them remain open to approaching situations and flexible to change, as problems are not always avoidable. In every stressful situation, there is growth, and by allowing ourselves to find opportunities for growth, we will attain practical skills to remaining calm and comfortable in stressful situations…”  30M: “…the benefits that I have reaped from meditation have allowed me to be productive with my work without having to get sucked in an unhealthy lifestyle…”  35F: “…resilience and mindfulness… are essential and important for me to do to help me with burnout and stress which are integral to becoming a future physician…”  36M: “…the transition between high school and medical school was a tremendous turning point in my life. Living away from my family, facing a new city, new curriculum system and difficult material were the major challenges. This turning point has created a huge stress on me and made studying efficiently hard. My initial coping mechanisms were social networking and talking to my colleagues. This worked in the beginning until I talked to one of the students from the previous batch who was repeating the year, which made me stressed again about failing the year and losing my scholarship. Since then, I spent more time studying and lost most of my social networking. My mental health at this stage was going through a hard time. Despite the time spent studying, my grades were not as good as I expected, and I thought I should bring this issue to my academic advisor. ‘Taking care of your mental health and social networking will definitely improve your grades and your satisfaction level’ my academic advisor said…”  37M: “…Some teaching methods, within medical school, are not ideal but we still have to go through them, and we need to utilize all resources available for us to avoid burnout. From my personal experience, resilience tools are excellent, and available for everyone, you might need some time to get to the state you want to achieve, but you will not get there if you do not try…” |
|  | Perceived requirement of conscious practice  **8** | 1M: “…developing resilience is a mental process very similar to developing muscles in our body… some individuals have these abilities naturally, with personality traits that help them remain unflappable in the face of challenge. However, these behaviors are not just inborn traits found in a select few individuals. I believe resilience is quite common, and people are capable of learning the skills to become more resilient… mindfulness can be as simple as being aware of your breath. Noticing the subtleties of your abdominal area as you inhale and exhale. It could also be about focusing on the food you are eating. Paying complete attention to the flavours, tastes, and spiciness…”  2F: “…resilience can be hard to practice consciously in our day-to-day lives, especially for a person with a busy schedule that has only recently started getting into it, as it requires a bit of reading and research to learn about the various ways to practice it, either through mindfulness, meditation, or positive reframing. However, I do believe that once a person knows what resilience is and how to practice it, the act of building it over time would take a few seconds to minutes of a person’s time, and it would allow them to feel more at peace…”  4F: “…I tried this for two weeks; I have set a plan to build my resilience by practicing mindfulness, prioritizing my relationships, and keeping things in perspective…”  7F: “…in a moment of stress, I thought about trying out a mindfulness activity, there in the moment…”  11F: “…I find that resilience is a strong and helpful trait that enables me to continue working hard no matter what difficulties I face. It allows me to stay emotionally stable during stressful events, such as exams or losing a loved one…”  12F: “…resilience is not a trait that we are born with; it is a set of skills that one learns to develop and build slowly over time. It is a skill that we improve throughout our life...”  16F: “…resilience is not just a buzz word, it is a way of life, if you can identify the stressors in your life and not just sit idle but really try to untangle this mess, you would actually feel lighter…”  24M: “…I hope to master mindfulness to proactively use it to face every day struggles that occupy my mental space. At the end, it is not the destination that determines success, but the small victories throughout the journey…”  31F: “…in every stressful situation, there is an opportunity for growth, and by leveraging those opportunities, we will attain practical skills while calmly maneuvering through the situation…”  36M: “…I started taking mental health seriously and looked for several options on the Internet. Praying, taking breaks, practicing hobbies, and mindfulness were mentioned but I was not aware of the importance of mindfulness at that time. Praying on time, playing football, going out with my friends, and spending more time with my family, without affecting my studies, were the initial changes in my lifestyle. My grades started improving gradually until I got one of the highest marks in the batch in one of the exams. I was so happy but still I thought something was wrong and I was not satisfied enough. I revised the lifestyle changes that I had recently made, and remembered mindfulness and what I read on the internet. I prepared mindfulness objectives, for each week, and tried achieving them day by day…” |
| Application | Concept (What?)  **9** | 1M: “…mindfulness is a tool to escape and be present at the same time…”  2F: “…a person can build resilience through acts of meditation and mindfulness…”  3F: “…the practice of mindfulness helped me achieve a state of mind concentrated on the present by observing the flow of inner thoughts and emotions. It helped me to develop myself by increasing my self-awareness. I now recognize my emotions and their triggers, and instead of burying my head in the sand as was my previous custom, I am now able to face my anxiety head-on…”  5F: “…mindfulness elicits that we take a break from our daily activities, or better described as incorporating a new daily activity, in which you try your best to bring all your attention and focus to the present moment. Mindfulness allows one to get in-sync with their breath. This helps to increase one’s awareness and enhances their state-of-mind…” 13M: “…… being mindful means having the ability to be aware of our surroundings, and not overreact or be overwhelmed… one must be mindful of what is surrounding them, as this will allow for early recognition and preparedness to face the adversity. Mindfulness also allows us to be aware of what is going on inside and around us, providing a clear picture of our situation; and this enables an individual to respond in a manner appropriate to each scenario…”  14F: “…it is almost magical how versatile mindfulness is, taking innumerable shapes and forms, and influencing our lives in diverse ways. It can be incorporated into anyone’s lifestyle, through particular activities, including ones that address physiological cravings and consequential behaviors…”  22F: “…being resilient and mindful is crucial to have a healthy ‘academics-life balance’… acknowledging that difficulties in life are inevitable and remaining mindful that everyone around us is facing some sort of difficulty in their lives helps me to tackle whatever situation I find myself in and enables me to work on ways on how to deal with such situations. To me, that is the first step in being resilient…”  28M: “…with respect to mindfulness, I have been able to understand a lot of insecurities and issues I had, which were leading to my troubled mind; it has also helped me understand how to deal with people and with situations I feel uncomfortable with…”  35F: “…as a person who has always doubted oneself and was my own worst enemy, I built resilience through silencing these voices of self-doubt… I built myself back up and started afresh…” |
|  | Exercise(s)/ Activities (How?)  **26**  Mistake: we reviewed this for appraisal of exercises. Correction: we will consider what we did as an extra layer of analysis (italic). We will be reviewing the same section again for ‘how’ (highlighted in Yellow).  How are we going to communicate the modification in the analysis method?  While reviewing the ‘how’ category, we noticed that it includes a lot of ‘exercise appraisal’, so we introduced an additional layer (indirect appraisal versus direct appraisal). | 2F: “…this can be done through simple activities, such as the five senses exercise, which could calm a person down during periods of anxiety. This not only allows the person to calm down but also to pay attention to the surrounding environment... another way to practice mindfulness is through building awareness of one’s own breathing …I choose to walk around, either at home or outside, allowing myself to let go of life’s stressors…”  *3F:* “…my first experience with mindfulness was *simply by observing my backyard garden. As familiar as I presumed myself to be with the scenery, I found myself noticing the simplest yet most intriguing details. For once, I dwelled upon the trees and greenery instead of the scorching hot sun of Dubai. It was astounding how focusing on such robust objects produced an immense amount of relief. Instantaneously, I could sense physiological changes within my own being and a shift of consciousness…”*  4F: “…my classmates and I, during one of the sessions, we were led to mindfully eat a date, for the first time…”  *6F: “…it is such an easy task, and it takes less than 5 minutes. It can turn around your entire day…”*  7F: “…the resilience course included a mindfulness tracker which was to be completed as a course requirement…”  *8M: “…one thing that I always do when I feel down is looking back at myself a few years ago and asking, ‘aren’t there many things that you wished for back then that you have now?’ This question never fails to make me feel better.* I wanted to get into medical school, I wanted to drive around in a car, and I wanted to stop spending so much time on video games and exercise instead… a simple exercise when I drive to the hospital. I roll down the windows, appreciate the weather in the morning and play a song I like… this may only work for me because I believe that it can work…letting go of things that may ruin my day, such as memories… that it is part of growing and it most certainly helps me with moving on with my life and being the best version of myself…”  *9F: “…practice of reflecting is also very crucial, it helps one to step beyond the situation and exercise viewing it differently, it gives you the ability to be a spectator…”*  *10M:* “…there are several methods in which one can build or establish resilience such as reframing one’s thoughts, in other words by not viewing a glass as ‘half empty’ but rather as ‘half full’, and focusing on what is within our locus of control... sometimes additional support from a friend, family member, or counsellor may also help in building resilience… some of the mindfulness exercises include focusing and paying attention to what is at hand, focusing on the present moment, accepting one’s self, and focusing on breathing, meditation, yoga, exercising… *I personally find several things that help me such as relaxing when I feel tired, knowing my limits, and not pushing myself beyond them to avoid burning out, time management, listening to music and sometimes just sitting in silence and peace for a few minutes may provide me with the break that I need to be able to continue.* Additionally, the support of family and friends is always vital…”  12F: “…simple acts of meditation and mindfulness like the five senses exercise can help us to adapt to the new environment we are in and to the new stressors we are facing by using our five senses to focus on our environment instead of our racing thoughts to help us calm our mind and let our body know that there is nothing to fear”  *14F:* “…stories regarding meditation, ‘time-outs’, and ‘tea breaks’ practices being soothing for the soul and strengthening for the will to tackle stressful days have been circulating for centuries… *similarly,* *I have, over the years, intuitively learned to slow down and re-read the situation with fresh insight to truly master any difficulty, and looking back at this now, I see mindfulness in this practice, even without having had an any formal education about it.”*  *15F:* “…after becoming more familiar with the concept of resilience, I moved to practicing it in my daily life. That was done by identifying stressors that I faced in medical school or life that led to reduced satisfaction from medical education causing burnout that affected my general health and lead to suicidal ideation. The stressors affecting me personally where circumstantial, family based and I felt that I was behind everyone when it came to academics. To prevent such negative effects from triggering a serious chain of events, I turned to a practice of meditation called mindfulness… through activities such as progressive muscle relaxation, body scan, and breath meditation. In progressive muscle relaxation, you tense a group of muscles as you breathe in, and you relax them as you breathe out. The concept is that when your body is physically relaxed, you cannot feel anxious. The body scan trains the mind to be more open and aware of sensory experiences, and ultimately, more accepting. *I have been using the method of breath meditation for years on my apple watch and I can attest to the fact that it has helped me profusely with providing an immediate sense of relaxation… relieve stress and be fully present in my life.”*  *18F:* “…there are many primary steps and strategies designed to help nurture mindfulness, such as, paying attention to each sense – vision, audition, olfaction, gustation, and taction; taking time out to focus one’s concentration on each part of the body – head to toe; and many more. The purpose of these methods is two-fold: to live in the ‘now’, which implies that the past has been accepted, and the future has been left alone to carve itself out when its time comes; and to appreciate the little things in life that one often takes for granted. The appreciation subsequently matures into gratitude; and gratitude matures into positive feelings that have the power to negate stressful thoughts brought on by adversity… meditation falls on the spectrum of mindful practices. It is one of the more meticulous and specialized forms of mindfulness. A variety of types of meditation exist, ranging from transcendental meditation, yogic meditation, progressive relaxation meditation, chakra meditation, and visualization meditation among many others – all sharing the same fundamental principles: to be able observe one’s thoughts from a neutral perspective. This directly encourages the meditator to let go of their present worries and simply observe them from a safe distance in their mind, which in turn allows them to see the situation for what it is, and respond appropriately, instead of succumbing to it or reacting to it based on situational negative emotions. *Meditation can be seen as being akin to bathing the physical body – it is the cleansing of the mind. When practiced in the morning and at night especially, it purifies the mind of worry and concerns, and allows one to start each day afresh like a clean slate”.*  19F: “…simple acts of meditation and mindfulness can help us in adapting to the new environment we are in and to the new stressors we are facing. These simple activities include the five senses exercise, which could help us calm our mind by using our five senses to focus on our external environment instead of our thoughts. It helps to let our body know that there is nothing to fear and helps put a stop to racing thoughts…”  *20F:* “…learning how to handle stressors better in the future can be done in various ways such as recharging before facing a new challenge, taking a break from routines, and by finding laughter in stressful situations. Many, including myself, find that religious or spiritual means help build resilience. It can influence how we live, cope with stress, and how we recover from emotional strain. *For me, leaning on faith and having that spiritual purpose means having peace of mind, stability, and the security of knowing that I have my own compass to guide me through anything…* there are vast ways to practice mindfulness. Simplest form of practice is being in tune with oneself and being fully aware of our breathing, thoughts, feelings, and sensations. *Traditional methods of contemplation can also be tools such as yoga and meditation but developing the awareness to one’s breath can truly be a game changer.* Mind wandering has also shown benefit despite everyone suggesting that one must be focused on the present, to be here and now, but we as humans have the unique ability to give freedom to our minds and divert our attention to something that might not be necessarily in the present. Whether it is being on our phones and reminiscing about previous holidays or even future ones, brief wonderment may bring peace and happiness…”  22F: “…whenever I feel stressed about studying and deadlines, I take a moment to reflect on the situation and why I find it stressful. Sometimes, it would be because of the lack of time, other times it would be due to the enormity of the tasks I need to complete and hence feel intimidated to start which automatically makes me feel demotivated. When I take a deeper insight into the reasons why I feel stressed, it makes it easier to handle the situation and enables me to tackle the situation with a less anxious mindset….on days when I succumb to my anxieties, I try my best to take a moment to be mindful of my situation and do something small such as disconnecting from my phone…..doing small everyday tasks like disconnecting from my phone is an act of mindfulness; it helps clear any distractions and enables me to stay focused on my goals for the day…”  23M: “…. the best way to do mindfulness is by solely taking the time to do the exact opposite of overthinking, which is not thinking at all. Through self-guided meditation I have developed many scenarios that work alongside the theme of the chaos and work its way back to a more suitable ‘mental space’. I found that my preferred metaphorical medicine is to visualize the chaos and control it. If you compare the human mind to a sugar-infused hyperactive monkey who just got bit by a scorpion, you will start by closing your eyes and slowing down your breathing. When you are ready, you will visualize this monkey and focus on what it is feeling, how it sounds, and its current surroundings. Once you are comfortable with that, you start to tackle the problem one at a time and bring peace into its mind and heart. So, you will first work on treating the scorpion sting, analyzing the scorpion and what it represents and eventually removing it from the premise. Then you will work on the hyperactivity by either giving the monkey something to focus on (something peaceful like slowing down its breathing just like you, after all it’s ‘monkey see monkey do’). Lastly, you will take away the sugar that has put too much energy into this monkey and give it some nice warm green tea to calm it down while it does nothing but breaths. Finally, you are left with a calm mind and a clear canvas, and once you leave the situation and return to oneself, you are ready to be mindful. Now, you can focus on slowly and neatly bring up symbolic displays of the goals you wish to achieve during your day, or nothing at all if you wish to sleep during the night…… I also use the analogy of a library (of thoughts) that has been overrun by a tornado and requires reorganizing. Although cleaning up and reorganizing might not be as peaceful to most people, I find this a wonderful analogy to help me repurpose my cognition into something more peaceful and productive, with every thought representing an idea I want to either work on or simply put away in an organized matter where I know where and when it is most appropriate to do so…”  *25F*: “...being mindful of the situation and seeking an active solution has been very effective in tackling a problem like burnout and exam stress. Therefore, I started preparing my material day by day to avoid leaving too much to study on the week before my exams. I also developed the ability to create organized study schedules that help me with time management. I started implementing extra-curricular activities like jogging and dancing to make myself more comfortable and less stressed. In addition, if these ‘problem-focused’ coping mechanisms do not get the job done and I still find myself stressed with lots of material to cover before an exam, I calm myself and try to focus on high yield topics rather than reading through everything vaguely and quickly. Furthermore, *I have found that having an appreciative approach to small details around me has been very helpful….* taking a walk outside is very refreshing and helps me reset my mentality and set focus on what needs to be done. I am also grateful that none of my family members were affected by the COVID-19 pandemic and that everyone is in good health. Moreover, during my journey as a medical student, I always come across challenges and obstacles that make me doubt myself and contemplate giving up. *I have found that taking a step back and comprehending what I have achieved so far in life* and how close I am to my goal has given me the perseverance and the resilience to go on. Looking up to accomplished doctors and setting them as role models, picturing myself in their shoes in the future never fails to give me the motivation and the drive to give it all…”  *26F:* “...I have been practicing how to effectively use my time by learning to strike a balance between work and leisure. By organizing my day and keeping a daily planner, I have been able to effectively prioritize long term and short-term goals by creating daily and weekly targets. Upon reflection, I have realized that I used to engage in various maladaptive coping mechanisms such as avoidance and denial. *By attempting to be more aware of my choices when dealing with difficult choices or situations I have been able to come out the other end more resilient than before…”*  27F: “…the practice of mindfulness helped me achieve a state of mind concentrated on the present by observing the flow of inner thoughts and emotions. It helped me train my behaviour by increasing my self-awareness. I recognized all of my emotions and their triggers, and instead of burying my head in the sand as was my previous custom, I am now able to face my anxiety head on…”  28M: “…I am going to reflect upon some specific instances where I have used mindfulness: while talking to people, if they have a different opinion, I am mindful about respecting and not dismissing their opinion. I am now more aware of when I am lying due to nervousness and impulsivity, and I try to ground myself to remain authentic/ truthful. Finally, while having a conversation or during class, I am aware when I am losing concentration…”  29F: “…one way I personally find useful is removing yourself from the situation or stressor to be able to calm down, basically unplugging yourself from the source of fuel. Taking a walk to organize your thoughts is something I particularly find useful or going to the gym to let off some of that built up steam…”  *30M:* “…apart from a lot of the breathing exercises and relaxation techniques, *one method that has greatly helped me has been body scan meditation…”*  33F: “…I had my fair share of battles that I had to face alone, and I came out of them with helpful skills. One of the many things that I do is reflect on the stress of trauma and think about what I could have done to avoid them or what I can do to cope with it, for example I study a week earlier for an exam so that I do not have to stress about not having enough time to study or if a friend of mine is upset with me I would go and talk to them to see what I did to upset them then reflect on my actions. Another thing I do, is that in stressful times, I walk away from the source of stress for a while and go pray or sit down somewhere far away and take a few deep breaths just to focus my mind on other things, for examples if a friend of mine says something that bothered me, I would walk away, sit somewhere and take deep breaths then I will approach my friend and tell them that what they said bothered me a little. Another thing that helped with coping with stressful situations where painting as well as exercising, as it gave me a more creative output and physical stress reliever… I practice mindfulness through activities that bring me joy, such as praying, exercising, painting, and knitting, I also try to stay positive as much as humanely possible and see the bright side of things even if they aren’t as good. It is also very important to me that I have adequate sleep and eat a variety of good foods to feed the mind, body, and soul…”  *34F:* “…mindfulness to me is focusing on the small things in life rather than the big picture, because that just adds to my anxiety and thought load. I try to focus on the day itself: the people I see, the conversations I have, the food I eat, the tasks to be done today only. The extent of my future planning is limited to the future assignments I need to get done, and any outings I want to plan. But they all are in the near future. Focusing on long-term plans can be very stressful, as you cannot control the circumstances, so I let that be. *I also try to get some yoga into my schedule even if once a month, just to feel refreshed and rejuvenated. It reminds me to listen to my body and take care of it.* Taking time for myself, if it is the 10 minutes of commuting to my next destination, or going for a walk around the hospital grounds, *all helps me stay relaxed and mindful of my blessings…”*  36M: “…Being mindful of my family, friends, medical school, scholarship, and hobbies was the main objective, and has changed my way of looking and things and life. After 1 month I got the only full mark in my batch in one of the exams and I considered it a new turn point in my university journey. This was my first full mark since high school…”  37M: “…the first time I tried meditating was in a hotel I went to with my family one weekend. My sleep schedule was bad and that was the first thing I wanted to fix so throughout the day I did all I could to exert my energy (swimming, weightlifting) so I can sleep through the night and wake-up early. Before going to bed I closed the light and sat on the floor like monk and played one of the videos and tried to clear my thoughts. I kept trying 3 to 5 minutes and went to sleep. It was hard keeping my head clear from all thoughts, but it was working for a few seconds. I decided to write my progress on a piece of paper when I get home and stick it on my door when I get home to keep me motivated to try for longer every day…” |
|  | Faced challenges (Why not?)  **11** | 6F: “…practicing mindfulness is something that a lot of people probably have on their daily to-do lists, but never get around to it because, they either do not believe it works or they are too busy with their lives to stop for literally just a minute to breathe…”  7F: “…the resilience course included a mindfulness tracker which was expected to be completed as a course requirement… When we were informed about this assignment, I wondered how I was going to maintain a tracker because I had never done anything of that sort before. As a result of this, it took me a while to start, and when I finally did, it felt more like something I had to do for a course rather than something I was doing for my own benefit…I tried meditating, in the past, but always felt like I was missing the point because I thought I could not stop getting distracted…”  9F: “…practicing mindfulness can be considered, by some, as ‘difficult’ and ‘a waste of time’…”  11F: “…I do not regularly practice meditation. Firstly, because I do not have time or do not remember, and secondly because I do not feel the need to practice meditation…”  20F: “…however, learning how to pay attention to my thoughts and tune in to my emotions without having clouded judgment towards myself proved to be quite a task. I found myself judging how I’ve handled particular situations or how quick I was to express an emotion during an argument, but there’s no right or wrong. Powering through and accepting that that’s how I will grow was the only way for me to truly be mindful which in turn would aid in strengthening my resilience.”  22F: “….I have had many days in which I needed to be resilient but found it easier to just succumb to the anxiety and not do anything about it”.  23M: “…I must admit, my methods are not ironclad and are not practiced as frequently as one needs (I call it the “maintenance dose”) I am working alongside my greatest enemy of productivity to better my chances of succeeding in both my personal and professional life…”  27F: “…To be frank, upon encountering mindfulness I was nothing short of skeptical and somewhat doubtful. How could the answer be simply staring or contemplating? Apparently, utilizing the most complex and grandest biologic frontier, the human brain, was too simple for me of an answer…”  28M: “…initially, when one starts with exercising mindfulness, it can be really tiring…”  31F: “…mindfulness is the non-judgmental awareness of our thoughts, feelings, sensations, and surroundings. Although, this is not the easiest of tasks that a person might assign themselves to, to practice mindfulness we must train ourselves to stop rehashing the past in our thoughts or imagining the future, and actually remain in tune with what we presently sense … learning how to pay attention to my thoughts and tune in to my emotions without having clouded judgment towards myself proved to be quite a challenging task; I found myself judging how I have handled particular situations, or how rushed and haphazard I was in expressing an emotion during an argument. It is all easier said than done…”  35F: “…resilience and mindfulness were things that I never really had the time for, but now they are coping techniques that help me during tough times…” |
| Appreciation/ Appraisal | Building resilience  **18** | 3F: “…to utilize my own consciousness to battle the many emotional challenges that there are to be experienced, is a healthy coping mechanism and an empowering way to declare independence. For that, I am grateful…”  6F: “…practicing mindfulness, especially during these challenging times, allows me to be calmer and more peaceful, and it makes me more aware of my emotions so that I can regulate them better… mindfulness is a key part of my weekly routine that helps me destress and start a new fresh week…”  7F: “…I think I want to make mindfulness a habit because I personally seem to get lost in situations. I usually forget to live in the moment and make the best of the present. I have noticed that practicing mindfulness tends to help me with this. Mindfulness helps me live in the moment and this makes me feel better…”  8M: “…being mindful of what I have achieved gives me peace of mind. These little thoughts make me feel grateful…”  11F: “…Often, I found that relaxing and meditating made me feel better when I could not identify a specific cause of my emotions”  14F: “…Personally, resilience has been a big reason why I am where I am today; I owe resilience the ability to successfully navigate parental divorce, war, immigration, new schools and a career in medicine. However, I have not been aware of how much my resilience has played a role in all these milestones. Looking back, I now see that if I was educated about mindfulness, meditation or coping mechanisms I might have navigated my life with greater ease and awareness…only recently have I come to significantly take more control over my wellbeing and interpersonal relationships, thanks to mindfulness. I have noticed that losing sight of the truth of the situation in front of you and getting trapped of what could possibly happen boils you from the inside out. Your fear of the future and your anger towards your present drives you out of control, and specifically through mindfulness you can truly learn to live these challenging moments without judgement or hastiness.”  16F: “…..I wouldn’t have learned that I’m capable of staying calm and can see the silver lining of this challenge…. I won’t deny the moments of vague darkness that I went through but I discovered that I can calmly analyze and figure out what was bothering me , and why I felt the way I felt…..it gave me a super power, which i used for my good...it gave me the opportunity to understand others, like for example; it is wrong to call someone a drama queen and dismiss their feelings without first asking them what is really wrong… I learnt to let go off the bags of sands that were holding me back, and just float to the surface. I feel I can breathe now.  17F:”…. resilience was and will forever be the reason I am who I’ve become…. resilience is I, and I am resilience”.  18F: “…given the immense potential of benefits that mindfulness confers upon its practitioners, I believe that everyone, (especially healthcare professionals), should allow the peace and calm of mindfulness to permeate their everyday lives”.  19F: “…practicing resilience through simple acts of mindfulness and meditation is essential in our life as it gives us the psychological strength to cope with stressful situations in our life.”  21F: “….I was fascinated by the changes that mindfulness had on the writer of the book ‘Into the magic shop’, so I started meditating every day for a full month. My personality started to change for the better, I was less stressed, I began to read and discover more about meditation and mindfulness… I had all 6 domains of resilience, and at that time I did not know that I gained all of this just because I practiced mindfulness for some time… the main domains that helped me during my medical school were as follows: starting with "vision", I always had a goal to myself even with the simplest tasks, I had a purpose in everything I was doing, which helped me to do my tasks with passion and much effort. The second is composure, I was able to cope with stress healthily, so being stressed did not affect my mental or physical health like it used to do, I faced many situations with a calmness which helped me understand and solve any problem that I face in life….the main advantage that I gained from resilience was how to manage my health, I started to understand my physical health better, exercising, and healthy eating were some of the major life changes that I have done, not only it has improved my physical health but also my mental health. Mentally I am in a stage that allows me to understand myself, and have empathy for people surrounding me, with healthy coping skills and good time management that I was able to learn from my mindfulness journey, I improved my mental health drastically”.  22F: “…when all else fails, I remind myself that it is okay to have moments where you feel defeated by stress, but it is important to get up and work harder to compensate for the time lost to regain yourself”.  25F: “…If I didn’t practice mindfulness, I think I would have burnt out very early during medical school and I would have had a very tough time navigating school and my personal/social life”.  27F: “…to utilize my own consciousness to battle the many emotional challenges there are to be experienced is a healthy coping mechanism and an empowering way to declare independence. For that, I am grateful…”  28M: “…last year I was diagnosed with major depression, and I would say mindfulness had a big part in helping me deal with it… at some point, it becomes a second nature where in a situation you can realize what is the best course of action and how one should handle it. It is thanks to mindfulness, it has become easier for me to take criticism and share my opinion…”  32F: “…I have become more flexible to withstand all the pressure that I am encountering in the hospital. I am currently doing my surgery rotation and enjoying every part of it, including my mistakes. I am not scared of making mistakes anymore as this is what enabled me to try new things and learn more each day. Sometimes, I get anxious, but I am usually able to deal with this anxiety and use it as a motivation to work harder the next time…practicing resilience also helped me in my daily life. It offered me an opportunity to look back and reflect on every single day. It made me enjoy the little details of everyday life, and most importantly, it enabled me to understand that anything that helps my mental health is just as significant as my studies and my rotation…I started finding time for myself to read a book, watch a movie, hang out with friends, sit with my family, go out for a walk alone, and many more activities….this is very surprising for me as I am usually the type of person who is always studying…. the time that I have allocated for myself has allowed me to have a clear mind, enabled me to focus on the present moment, and carefully plan my next step…”  33F: “…having good resilience skills can help in facing all battles and helps to make their scars less painful… having resilience and being mindful is not only important in my personal life but also in my work life as it will help me stand up every time I feel like a failure. ….it will help me approach and help people in a better manner as well as have empathy towards their battles, whether physical or mental through mindfulness and resilience…”  34F: “…The importance of resilience and mindfulness cannot be denied or overlooked, especially in a field like medicine. After years of training, studying, and practicing in the field, it is essential for everyone to gain good coping skills if they wish to maintain a good mental health…” |
|  | Course  **14** | 4F: “…when starting the resilience classes, I realized how easy it is to do and that it is not time-consuming. When Dr. Bhavna was teaching us resilience, the information was easy to grasp, and the exercises were not emotionally and physically challenging; instead, it was very soothing. Besides, I expected to come into this class only to learn different meditation techniques; however, I usually left these sessions with learning the theoretical knowledge, processes, and outcomes of practicing mindfulness. All the points covered in the sessions were backed up by research, which made me eager to start and change the way I live my life daily…. I also realized that I was not the only one going through a rough time mentally, which made me feel less alone. Also, at the end of each session, I always felt good and relaxed…”  6F: “…having this as an actual assignment was exactly what I needed to get a jumpstart on mindfulness techniques in my daily life…”  7F: “…this course helped me learn and practice resilience skills like mindfulness…I realized I started to feel better after that. If this is the only thing, I got out of the resilience course then I think the goal of the course was achieved for me…the resilience course also motivated me to start writing a journal. It is still a learning experience for me because I sometimes feel like I do not know what to write but I have noticed that journaling helps me feel better and is a way for me to sometimes reflect on events or on my day and that in of itself makes me feel good…the resilience course made me learn that meditation is not about not getting distracted at all, but it is about bringing yourself back to the moment when you get distracted. This made a big difference for me and though I seem to do more mindfulness than meditation, I do not feel like I am failing at it completely, like I did in the past. I look forward to making this a regular part of my life and I hope it makes me a more resilient person than I already am…”  8M: “…discussing the way I perceive life and its changes and how I deal with it has been enlightening during this course. I look forward to improving my mindfulness and implementing more of it into my life, as time passes…”  11F”:… One thing I learnt from this course is to accept my negative emotions, like anger, anxiety, and stress, and acknowledge them. Once I started acknowledging my emotions, I started looking for the cause of those emotions to solve them….. I am convinced that mindfulness is beneficial for many reasons, and I needed a push to apply what I learnt in the resilience course to my daily life.  14F: “…I truly believe in the importance of resilience, and it is only through this course that I have been exposed to the different methods of strengthening this prime skill.”  15F: “…during the longitudinal theme course, we were able to examine the phenomena of resilience in much-needed detail. It all started with the introduction of the concept of mindfulness which has great all-around evidence-based effects…. by examining different coping mechanisms to increase our own personal resilience, this course has managed to aid us in coveting a skill that is essential in life.”  16F: “…I’m glad I attended the weekly sessions, and even though I was the biggest skeptic of this emotional mumbo jumbo, I took the quizzes- full disclosure they were fun-, they gave me an insight that I’m adapting, implementing and influencing…..I just hope we had more sessions, and they would be tailored to us….at the end the only thing I can say is…THANK YOU.  26F: “….the resilience course made me far more aware of the way I have been dealing with life all these years. My interactions with people around me, my coping mechanisms with stress and workload, what I do when I feel down and what things I resort to when I need to feel happier. These things often go unnoticed to most people. However, out of experience, I now know that ignoring my own state of mind when life throws everything at me tends to take a huge toll on my mental health, motivation, and overall happiness”.  25F: “…this course was delightful, it made my colleagues and I more open to constructive thinking…it pushed me further into figuring out solutions to my everyday problems and struggles…. it inspired me to be more thankful for the good things around me and less judgmental of the things I think are bad. I also realized that resilience is part of everyone’s life. It presents itself in the form of actions and habits that we do on a day-to-day basis. And we can always improve our habits, actions, and the way we look at life if we are more mindful of the way our minds interpret things we encounter.”  26F: “…the weekly mindfulness session held by the university has been able to point me towards the right path when trying to decide how to cope with my daily struggles. I have usually not been a person who could sit in one place and contemplate the events that have occurred during the day. Furthermore, I am usually very anxious and worry about the future quite often. While learning to be aware, I have attempted to take out 5 minutes of my day in the morning or evening to center my focus and try to concentrate on the present moment rather than worrying about what has happened or is yet to happen. I have achieved this by combining various techniques such as meditation and muscle relaxation. I have also learned to focus on my breathing when I feel my mind starting to wander. While all of these skills have helped me learn how to stabilize my mental state, another key aspect that caught my interest during the mindfulness sessions was the idea of gratitude. People usually take their life and surroundings for granted. However, working in the medical field, I have been able to observe how people are affected when these things may be taken away, such as the loss of a loved one or being diagnosed with a chronic illness. Seeing this on a daily basis has made me learn to be more aware of the small things in life that I may have taken for granted before. I have also learned to look at the positive things that happen in my life instead of just focusing on the negatives. I have created a small jar in which I add in one thing I may be grateful for during that day, and when I have bad moments or feel low, looking at those messages have been able to make me change my perspective about negative events. This technique has also made me aware of the overgeneralization of negative occurrences over positive ones. Overall, this course has helped guide my journey of improving my own mental health which I am hoping will make me a better healthcare professional…”  30M: “…It was at the start of my 4^th^ year when I was exposed to many different resilience techniques which have been able to calm me down and help me regain focus…”  32F: “…I have not been able to put it into practical use until I had some practice during the resilience course…. this was of utmost importance, especially during my rotations, which have caused my anxiety and stress to increase a lot. During the first few weeks of my rotation, I was always worried about how I am performing. I had little confidence in my abilities and was scared most of the time that I am not meeting the expectations of the doctors. Thus, during that time, every little mistake I did would put me in a very bad mood for few days and would make me doubt everything I know. As a result of that, I was not able to learn anything from my mistakes; they represented bad experiences rather than opportunities to identify my weak points and work on them. However, this mentality has changed drastically because of the resilience course…”  35F: “…I am fortunate to have had the opportunity to be part of this course, and in turn create all of these memories and learning experiences that I will never forget… this course gifted me new coping skills that can be done at any time to cope with every challenge I face, and come out stronger…overall, one of the things that I am truly grateful for was definitely this course. It gave me a new perspective, effective problem-solving skills, made me more aware of my surroundings and helped me let go of everything that was a barrier between me and my goals. It gave me the strength to wake up every day and attend to my duties and responsibilities not just as a student, but as a friend, daughter, sister and a human…” |
|  | Exercise(s)  **12** | 4F: “…my classmates and I tried mindful eating for the first time using a date…during the exercise I was asked to use all my senses while eating this date and to chew thoroughly and slowly. That was a pleasant experience that helped bring my attention to the present and simply indulging in the sweetness and texture of the date…it was difficult for me to be mindful when experiencing thoughts concerning my anxiety. I would explore my anxiety by asking questions like: ‘why am I feeling this way?’ and ‘is it caused by a problem that I can manage?’… questioning myself did not always help, but I will not give-up on this exercise. I know that, in the long run, it could improve the quality of my life… resilience was a foreign concept to me that seemed very farfetched but once I got to learn what it was it opened my eyes to how life changing it can be. Practicing mindfulness in my life has brought me a lot of comfort as it helped with my anxiety and my overthinking tendencies…”  7F: “…It also helps me to be grateful for even the ‘little’ things around me and in my life. I also find that I feel more balanced and can better respond to situations around me…I wish I started practicing mindfulness earlier but if there is one thing I have come to know about life, it is the fact that it is never too late...”  11F: “….I try to spend 5 minutes at the end of the day before I sleep to count my blessings and thank God for all of them. I love saying “Alhamdulillah, not only because of the Islamic value, but also because it is a reminder for all the blessings I have in life….. I sit in my garden, and just focus on one thing, usually the sound of the birds. I feel that it is refreshing and allows me to divert my negative thoughts away… I tend to prefer problem focused coping skills such as creating a to-do-list and goal setting. However, I recently found that emotion focused coping skills are helpful. I started accepting temporary failure such as a bad exam and talking to a friend whenever I feel low.”  15F: “….at the request of our mentor of the resilience sessions, I have started using a reflective journal wherein I used it to be mindful and grateful everyday.”  20F: “…I have personally found that mindful breathing and prayer help achieve inner peace where I can genuinely be centered in myself. I have had to power through practicing mindfulness which is vital in order to be resilient. I have come to learn that practicing mindfulness involve acceptance and that being non-judgmental…”  23M: “…I have preached to many of my fellow classmates about the importance of spending time with oneself and their surroundings, dismissing any lingering thoughts that may come to ruin a beautiful moment spent, a walk, reading a book, or even enjoying a song. As a person who practices what he preaches, I have opened myself to many new experiences that allows me to become one with the moment, such as enjoying a walk, doing some Tai Chi, and even deleted my social media accounts to limit unwanted mindless scrolling and procrastination…”  25F: “…my workdays are usually fast-paced, but practicing mindfulness allows me to slow down for a moment. I take a second to take a deep breath and I let my thoughts “slip” – I do not think of anything, and I sit in silence for about 5 minutes. I do this at home usually after I have finished an afternoon prayer. This has helped me clear my mind and I find it very relaxing. Sometimes I even do this on my commute to the hospital, or other times, I do it after breakfast or dinner…. it is enough to stop me from overthinking and getting overwhelmed with whatever I have running through my mind….it helps me cope with the stress… I enjoy practicing mindfulness in the morning right after waking up. I soak in all the sunshine from my bedroom window and I close my eyes, letting my thoughts go for about 10 to 15 minutes. This again has been a great exercise for me as it helps me embrace the new day. Another way I practice mindfulness is by working out. Running has always been a great stress-reliever for me as I feel like I am running away from negative thoughts and towards better ones. I also enjoy the accomplished feeling I get after a run (which is most likely due to the post-workout endorphin rush)…”  30M: “…although I cannot fully attribute this change to my practice of Resilience, I do believe that I have been a much kinder, calmer, and a more patient person since I had started using mindfulness and meditation as an outlet of releasing stress…”  34F: “…Learning about problem and emotion-focused coping mechanisms helped me see the different options around this. It is true that sometimes you can’t change the stressors in your life, especially right there in the moment, so learning to deal with it helps me focus on the present, ground myself, and enhance my productivity… Mindfulness has taught me to acknowledge with greater appreciation the present moment as is without any expectations and stress. I try to give myself credit for every accomplishment, no matter how small, instead of focusing on all I have not achieved. It has helped me become more aware, more mindful of every moment of my life, and has helped me increase my ability to regulate my emotions as well as decrease the amount of daily stress I deal with. In addition to how I view myself and my life, mindfulness has also improved my emotional intelligence and my ability to relate to others with kindness, acceptance and compassion. Overall, I hope I can continue to use these different techniques in my daily life, and learn to better focus on my mental health going forward...”  35F: “…At first, I was listening to these sessions while trying to keep up with our clinical rotations and the assignments that we are supposed to finish and submit by the end of the week. Later on as burnout and fatigue started to knock me down while sitting in the library, I thought why not listen to these sessions and see what resilience is all about. So, paid attention to what was being said just to change the scenario and decided to try one of the exercises that were being performed. The exercise was quite interesting, it was eating a date and how to be aware of all of your senses while doing so. I remember I didn’t have any dates with me but I imagined eating it and feeling it with all of my senses, and that started to shape my perspective on mindfulness and meditation. Another exercise aimed to highlight and convey certain things to our attention that we deem granted, like our legs and toes. Luckily, I managed to try this exercise, and my whole perspective changed. It made me realize that I take a lot of things for granted, especially after this year. It even made me appreciate the things that hurt me during that year which I thought I never would. On my way back home, while driving, I was able to think about the list of things that I was grateful for and managed to come out with a good number of things that kept increasing every time I meditated. I started to bring unconscious thoughts to life while performing day to day tasks even during assisting in surgeries during my surgery rotation, and talking to patients in my psychiatry rotation…”  36M: “…After seeing all these changes, I expanded my mindfulness objectives and had a specific time daily before going to bed as a mindfulness session. I started being mindful of not only the big details but the small ones, as well. The small details were things like perfume smell, food taste, bed, and night walks. I was not facing anything without being mindful of its presence. My satisfaction level was a hundred times double that level I had when I first entered medical school. Throughout this long journey, praying on time and reciting Quran in addition to mindfulness were the source of assistance I run to whenever I face difficult time. I have never missed my mindfulness sessions since then, and I do not think I will be missing any mindfulness session in my life…”  37M: “…I did that for about 2 weeks and my sleep was schedule was adjusted and I started to worry less about the future. Meditation is a helpful skill that did work for me when I needed it…” |
